# The effect of physical therapy and mechanical stimulation on dysfunction of lower extremities after total pelvic exenteration in cervical carcinoma patient with rectovesicovaginal fistula induced by radiotherapy: a case report

**DOI:** 10.1186/s13256-024-04516-0

**Published:** 2024-04-13

**Authors:** Wujian Lin, Bing Yao, Jiahui He, Shuangyan Lin, Yafei Wang, Fangting Chen, Weichao Zhang, Jiashu Yang, Zhihong Ye, Jianguang Qiu, Yuling Wang

**Affiliations:** 1 Department of Rehabilitation Medicine, The Sixth Affiliated Hospital, Sun Yat-Sen University, No.26 Yuancun Erheng Road, Guangzhou, Guangdong China; 2 Department of Urology, The Sixth Affiliated Hospital, Sun Yat-Sen University, No.26 Yuancun Erheng Road, Guangzhou, Guangdong China; 3 Department of Rehabilitation Therapy Technology, Lvkang Bomei Rehabilitation Hospital, Ningbo, Zhejiang China; 4 Department of Rehabilitation Medicine, Foshan Women and Children Hospital, Foshan, Guangdong China; 5 Department of Rehabilitation Medicine, The Fifth Affiliated Hospital, Jinan University, Heyuan, Guangdong China; 6 Department of Rehabilitation Medicine, Fujian University of Traditional Chinese Medicine, Fuzhou, Fujian China; 7 Guangdong Provincial Clinical Research Center for Rehabilitation Medicine, Guangzhou, China; 8 Biomedical Innovation Center, The Sixth Affiliated Hospital, Sun Yat-sen University, Guangzhou, China

**Keywords:** Physiotherapy, Cervical cancer, Total pelvic exenteration, Postoperative complications, Case report

## Abstract

**Background:**

Total pelvic exenteration is the ultimate solution for rectovesicovaginal fistula caused by radiation therapy, yet total pelvic exenteration frequently causes intraoperative complications and postoperative complications. These complications are responsible for the dysfunction of lower extremities, impaired quality of life, and even the high long-term morbidity rate, thus multidisciplinary cooperation and early intervention for prevention of complications are necessary. Physical therapy was found to reduce the postoperative complications and promote rehabilitation, yet the effect on how physiotherapy prevents and treats complications after total pelvic exenteration and pelvic lymphadenectomy remains unclear.

**Case presentation:**

A 50-year-old Chinese woman gradually developed perianal and pelvic floor pain and discomfort, right lower limb numbness, and involuntary vaginal discharge owing to recurrence and metastasis of cervical cancer more than half a year ago. Diagnosed as rectovesicovaginal fistula caused by radiation, she received total pelvic exenteration and subsequently developed severe lower limb edema, swelling pain, obturator nerve injury, and motor dysfunction. The patient was referred to a physiotherapist who performed rehabilitation evaluation and found edema in both lower extremities, right inguinal region pain (numeric pain rate scale 5/10), decreased temperature sensation and light touch in the medial thigh of the right lower limb, decreased right hip adductor muscle strength (manual muscle test 1/5) and right hip flexor muscle strength (manual muscle test 1/5), inability actively to adduct and flex the right hip with knee extension, low de Morton mobility Index score (0/100), and low Modified Barthel Index score (35/100). Routine physiotherapy was performed in 2 weeks, including therapeutic exercises, mechanical stimulation and electrical stimulation as well as manual therapy. The outcomes showed that physiotherapy significantly reduced lower limb pain and swelling, and improved hip range of motion, motor function, and activities of daily living, but still did not prevent thrombosis.

**Conclusion:**

Standardized physical therapy demonstrates the effect on postoperative complications after total pelvic exenteration and pelvic lymphadenectomy. This supports the necessity of multidisciplinary cooperation and early physiotherapy intervention. Further research is needed to determine the causes of thrombosis after standardized intervention, and more randomized controlled trials are needed to investigate the efficacy of physical therapy after total pelvic exenteration.

## Introduction

Radiation therapy, including external beam radiotherapy and brachytherapy, remains one of the most important treatments for cervical cancer [1]. The use of therapeutic radiation has brought improved disease control and overall survival [2], but its effectiveness, which kills cancer cells, also destroys healthy tissues, causing side effects, such as rectovesicovaginal fistula, which is perhaps the most severe and disabling type [3]. The side effects result in a burden that affects quality of life of cancer survivors even for many years after the end of treatment [2]. The fistulae caused by radiotherapy remain a particular challenge, and are closed in only a third of cases [4]. Rectovesicovaginal fistula caused by radiation is often hard to repair, but total pelvic exenteration (TPE), urinary diversion, and digestive diversion coexist effectively, being the ultimate solution [5]. However, despite improved surgical technique and perioperative care, debate exists regarding the high rate of complications for TPE. It has a reported 2–14% operative mortality rate and 33–75% long-term morbidity rate [6]. The intraoperative complications include lymphatic duct, iliac vascular, and obturator nerve injury, which are responsible for dysfunction of lower extremities [7]. The postoperative complications include lymphatic fistula, lymphatic cyst, intestinal obstruction, pelvic hematoma, infection, deep vein thrombosis (DVT) of lower limbs, pulmonary embolism, and so on. [8]. Physical therapy can reduce postoperative complications and promote postoperative rehabilitation. In clinical practice, physical therapy is often applied in perioperative patients, and its effects are widely studied and reported in orthopedic surgery but lacking in total pelvic exenteration.

We present below a cervical cancer patient complicated with rectovesicovaginal fistula caused by radiation who received TPE and subsequently developed severe lower limb edema, swelling pain, obturator nerve injury, and motor dysfunction, which were significantly improved by physiotherapy and mechanical stimulation. The purpose is to investigate the effect of physiotherapy and mechanical stimulation on postoperative complications after TPE combined with pelvic lymphadenectomy and to provide evidence for multidisciplinary cooperation and healthcare management.

## Case presentation

A 50-year-old Chinese woman was admitted to the department of urology of the Sixth Affiliated Hospital of Sun Yat-sen University on 13 July 2020. She gradually developed perianal and pelvic floor pain and discomfort, right lower limb numbness, and involuntary vaginal discharge owing to recurrence and metastasis of cervical cancer more than half a year ago. Before admission, she could finish all daily activities herself at home, which meant that her functional status was satisfactory. Clinical diagnoses included rectovesicovaginal fistula, right ureteral obstruction urinary tract infection, and cervical carcinoma; she received external beam radiotherapy to the pelvis plus brachytherapy. On 22 July 2020, she accepted laparoscopic total pelvic exenteration, pelvic lymphadenectomy, sigmoidostomy, and radical cystectomy with construction of Bricker bladder, the whole procedure lasted 10 hours. On the first day after surgery, to prevent the formation of venous thrombosis, the doctor prescribed the appropriate dosage of low molecular heparin and anticoagulant drugs, such as rivaroxaban, on the basis of the results of Caprini score for venous thromboembolism and blood clotting tests. After surgery, the patient developed depression edema, pain, sensory dysfunction, and movement limitations in both lower extremities. Therefore, she was referred to a physical therapist on the first postoperative day and began physical therapy evaluation and intervention.

### Rehabilitation evaluation

Time for evaluation: postoperative day 1 (DPO1), postoperative day 7 (DPO7), postoperative day 14 (DPO14), as well as the day of discharge.

The main results of DOP1 were detected as follows: leg circumference was 39.5 cm (35 cm for the left) at the widest point of the right calf, the right ankle circumference was 24.5 cm (21.8 cm for the left); obviously observable edema appeared in the right lower extremities and pitting edema existed in both lower extremities; numeric pain rating scale (NRS) score of the right inguinal region was 5/10, pain increased to NRS score 7/10 at 35° right hip passive flexion with right knee extension; sensory function assessment revealed a decrease in temperature sensation and light touch in the medial thigh of the right lower limb, suggesting a skin sensation disorder; manual muscle test (MMT) is a grading system of muscle strength including 0, 1, 2, 3, 4, and 5, where 0 indicates no muscle contraction identified with palpation, while 5 indicates that muscles can drive the joint across full range of motion (ROM) and against full resistance applied by the investigator. The MMT with the patient in supine position discovered that the MMT of right hip adductor muscle was grade 1 and the right hip flexor muscle with knee extension was grade 1, which indicates dysfunction of the adduction and flexion muscles of the right hip joint; the ROM assessment found the patient unable actively to adduct and flex the right hip with knee extension, suggesting limited active ROM of the right hip; the total score of the de Morton Mobility Index (DEMMI) was 0; the score of the Modified Barthel Index (MBI) was 35, indicating severe dependence (dependent in mobility and in self care) and it was unlikely that the patient would be discharged and allowed to return home.

### Physical therapy

Timely and early physiotherapy to postoperative patients effectively reduces postoperative complications and promotes rehabilitation [9]. Therefore, according to the evaluation results of the patient in this case, we formulated the postoperative physical therapy program. The aims of physiotherapy designed by experienced physiotherapist were to reduce the edema of both lower limbs, relieve pain, enhance the sensory function of the medial femoral skin, improve muscle strength and active ROM of the right hip joint, and develop motor function and the activities of daily living (ADL), while preventing postoperative complications and controlling the length of hospital stay. The following shows the three main interventions: exercise therapy, mechanical stimulation, and electrical stimulation as well as manual therapy.

#### Exercise therapy

**Fig. 1:**
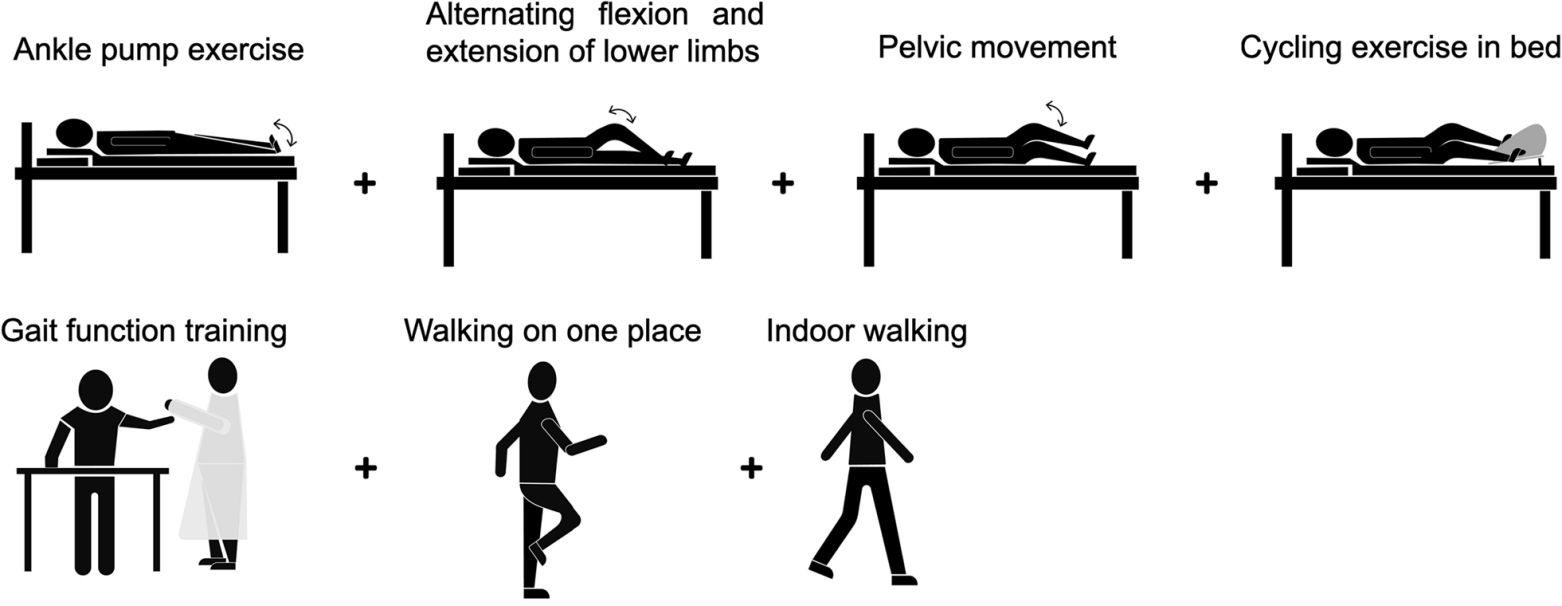
The standardized therapeutic exercises

Therapeutic exercises were tailor-made with a specific type of exercise, standard procedure of movement, intensity as well as frequency and time (Table 1 and Fig. 1). In addition, both position transfer training and ADL training were interspersed in the treatment process, with the purpose of enhancing the overall motor function, improving the ADL ability, and preventing the occurrence of complications.

**Table 1:** The standardized therapeutic exercises specifically designed for the individual

Type of exercise	Standard procedure of movement and intensity	Frequency and time
Ankle pump exercise [25]	Position: supine with slight abduction and external rotation of the hips to relax the thigh muscles, the knees and ankles are rested on a small cotton pillow Movement: maximally hook the foot (the toes face herself), and then stretch the foot (the toes go away from herself), sustained for 3 seconds at the end of the movement in the two directions	10 times/set, three sets/day
Alternating flexion and extension of lower limbs	Position: supine Movement: ipsilateral hip, knee, and ankle flex to the maximum, while the contralateral extend to the maximum, and then reverse contemporarily. During the early period the therapist facilitates hip flexion with both hands offering assistance	10 times/set, three sets/day
Pelvic movement	Position: supine with the hips and knees flexed to the maximum but heels touching the floor Movement: move both lower limbs from one side to the other side together in the same direction	10 times/set, three sets/day
Cycling exercise in bed	Position: supine with the feet on the pedal of the bicycle Movement: actively pedal the bicycle embedded in ward bed with minimal resistance	20 minutes/day
Gait function training	Position: standing in the walking frame Movement: reach for objects in all directions and keep balance	5 minutes/time, two times/day
Lower extremities weight-bearing training	Position: standing with the walking frame as a protection facility Movement: alternately standing on one leg	5 minutes/time, two times/day
Walking in place	Position: standing with the walking frame as a protection facility Movement: walk in place and alternately swing both upper limbs	5 minutes/time, two times/day
Indoor walking	Position: standing Movement: walking in the corridor near the ward	20 min/time, two times/day

#### Mechanical stimulation and electrical stimulation

**Fig. 2:**
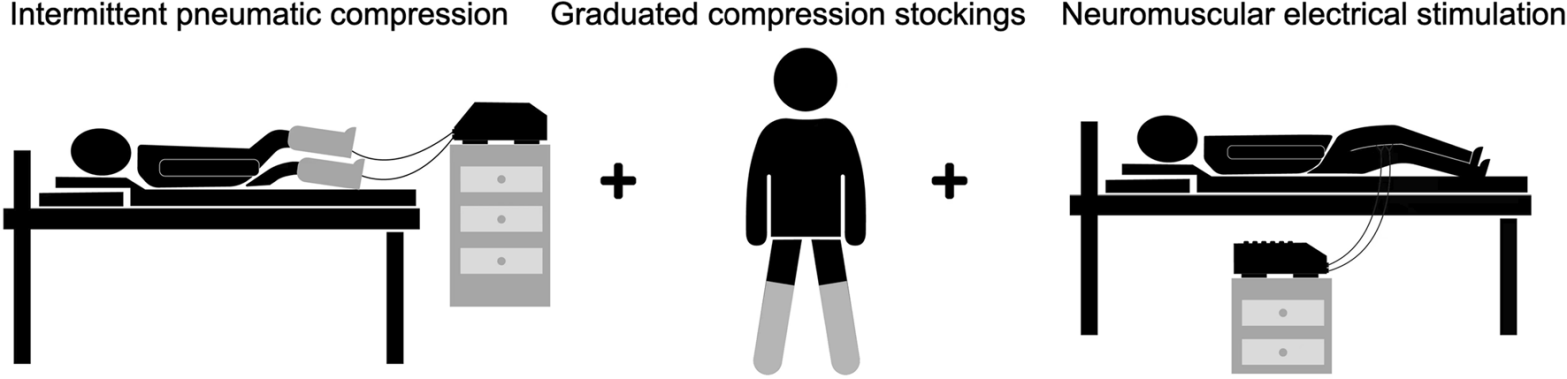
The mechanical and electrical stimulation

Table 2 demonstrates the mechanical stimulation including intermittent pneumatic compression (IPC) and graduated compression stockings (GCS). Moreover, neuromuscular electrical stimulation (NMES) was also applied to manage various clinical conditions (Fig. 2).

**﻿Table 2:** The mechanical stimulation and electrical stimulation

Treatment	Specific prescription	Effectiveness
Intermittent pneumatic compression (IPC)	The balloons enveloped both lower limbs in a distal to proximal mode with an intensity of 8 kPa and a duration of 20 minutes, using the compression device (LGT-2200WM, Longest Science & Technology Co., Guangzhou)	Pharmacological prophylaxis combined with IPC can reduce the incidence of deep vein thrombosis (DVT) more significantly than alone [26]. IPC is frequently applied in the management of lymphedema for complex decongestive therapy (CDT), which is particularly beneficial to those with compromised mobility [27]
Graduated compression stockings (GCS)	Wore for about 8 hours every day and took off when she fell asleep at night	GCS demonstrate the ability to reduce edema; moreover, GCS combined with low molecular weight heparin can significantly weaken the formation of venous thrombosis [12, 13]
Neuromuscular electrical stimulation (NMES)	Two pairs of electrodes were placed on the adductor muscle of the right lower extremity for 20 minutes a day, using a neuromuscular electrical stimulator (Hwato SDZ-III, Suzhou JSF Trading Co., Ltd., Jiangsu)	Studies have shown that NMES improved muscle strength, increased range of motion, reduced edema, reduced atrophy, healed tissue, and relieved pain [28]

*CDT* complex decongestive therapy; *DVT* deep vein thrombosis; *GCS* graduated compression stockings; *NMES* neuromuscular electrical stimulation; *IPC* intermittent neumatic compression

#### Manual therapy

**Fig. 3:**
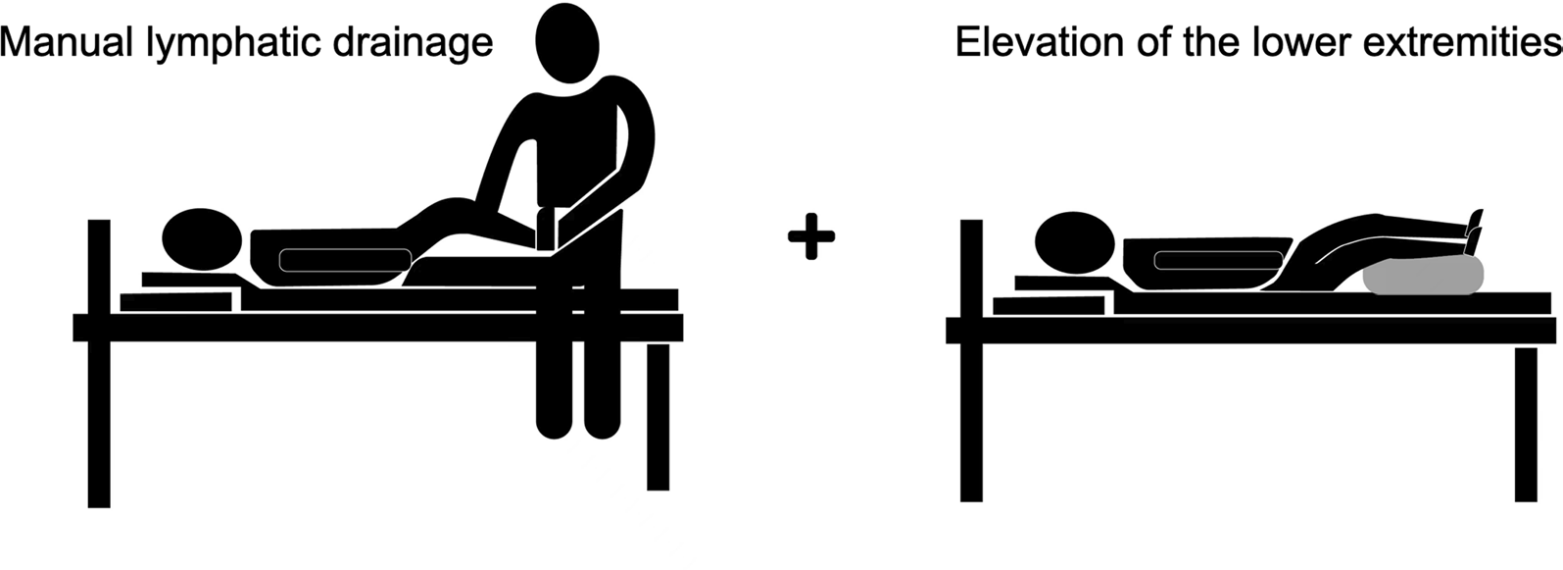
The manual lymphatic drainage and elevation of the lower extremities

Manual lymphatic drainage (MLD) massage remains widely accepted as a conservative treatment for lymphedema (Fig. 3). It is thought to soften fibrosis and facilitate lymph drainage into venous circulation by stimulating superficial lymphatic contraction and rerouting lymphatic fluid into adjacent healthy lymphatic systems; thus, MLD may help prevent lymphedema after cancer surgery [10]. MLD combined with elevation of the lower extremities by placing a pillow under the leg was also applied to the patient; this method has been described as having a good effect on reducing edema of the lower limbs [11]. In the case of exposed skin, the therapist fixed the ankle joint with one hand and the other hand gently massaged from the medial malleolus to the medial side of the calf, along the path of the lymphatic duct for 3 minutes/time, two times/day for each side of the limb.

### Outcomes

After 1 week of treatment, the edema of both lower limbs was alleviated. The circumference of the right calf decreased from 39.5 cm to 35.6 cm, and that of the left calf decreased from 35 cm to 34.7 cm, the right ankle decreased from 24.5 cm to 23 cm and the left ankle decreased from 21.8 cm to 20.4 cm (Table 3). The ROM of the right hip increased, the active adductive range of motion increased from 0° to 10°, and the hip flexion increased from 0° to 5° under knee extension (Table 4). The muscle strength of the right lower limb around the hip joint increased slightly, with the adductor muscle from MMT increasing from grade 1 to 1+ and the flexor muscle from MMT grade 1 to 1+ (Table 5). The pain was also relieved slightly, the NRS score of right inguinal region decreased from 5/10 to 4/10 and hip flexion and knee flexion decreased from 7/10 to 6/10 (Table 6). The overall score of DEMMI increased from 0 to 67 (Table 7), and she still had difficulties in static equilibrium and dynamic equilibrium. The MBI score increased from 35 to 68 and the ADL improved to moderate dependence (Table 8).

**﻿Table 3:** Limb circumference at different time points

Time	Calf		Ankle joint	
	Right (cm)	Left (cm)	Right (cm)	Left (cm)
DPO1	39.5	35	24.5	21.8
DPO7	35.6	34.7	23	20.4
DPO14	34.5	33.5	21.3	19.1

*cm* centimeter; *DPO1* Day 1 postoperative; *DPO7* Day 7 postoperative; *DPO14* Day 14 postoperative

**Table 4:** Range of Motion of the right hip at different time points

Time	Adduction (°)	Hip flexion (knee extended; °)
DPO1	0	0
DPO7	10	5
DPO14	15	5

*DPO1* Day 1 postoperative; *DPO7* Day 7 postoperative; *DPO14* Day 14 postoperative

**Table 5:** Muscle strength at different time points

Time	Adductor muscle group	Hip flexor muscle group (knee extended)
DPO1	1/5	1/5
DPO7	1+/5	1+/5
DPO14	1+/5	1+/5

*DPO1* Day 1 postoperative; *DPO7* Day 7 postoperative; *DPO14* Day 14 postoperative

**Table 6﻿:** The numeric pain rating scale of the right inguinal region at different time points

Time	Rest	Activity
DPO1	5/10	7/10
DPO7	4/10	6/10
DPO14	3/10	4/10

*DPO1* Day 1 postoperative; *DPO7* Day 7 postoperative; *DPO14* Day 14 postoperative

**﻿Table 7:** The de Morton mobility index at different time points

Activities	DPO1	DPO7	DPO14
Bridge	0	1	1
Roll onto side	0	1	1
Lying to sitting	0	1	2
Sit unsupported in chair	0	1	1
Sit to stand from chair	0	2	2
Sit to stand without using arms	0	1	1
Stand unsupported	0	1	1
Stand with feet together	0	1	1
Stand on toes	0	1	1
Tandem stand with eyes closed	0	1	1
Walking distance	0	2	2
Walking independence	0	2	2
Pick up pen from floor	0	0	0
Walks four steps backwards	0	1	1
Jump	0	0	0
Raw score	0/19	16/19	17/19
DEMMI score	0/100	67/100	74/100

*DEMMI* de Morton mobility index; *DPO1* Day 1 postoperative; *DPO7* Day 7 postoperative; *DPO14* Day 14 postoperative

**Table 8:** The modified Barthel index at different time points

Time	Personal hygiene	Bathing	Feeding	Toilet control	Dressing	Bowel control	Bladder control	Stair climbing	Chair/bed transfers	Ambulation	Total score
DPO1	5	0	10	0	0	10	10	0	0	0	35/100
DPO7	5	3	10	0	0	10	10	0	15	15	68/100
DPO14	5	3	10	2	5	10	10	5	15	15	80/100

*DPO1* Day 1 postoperative; *DPO7* Day 7 postoperative; *DPO14* Day 14 postoperative

After 2 weeks of treatment, the functional status improved. The edema of both lower limbs was alleviated significantly. The circumference of the right calf decreased from 39.5 to 34.5 cm, the left calf decreased from 35 to 33.5 cm, the right ankle decreased from 24.5 to 21.3 cm, and the left ankle decreased from 21.8 to 19.1 cm (Table 3). The ROM of the right hip increased, the active adductive range of motion increased from 0° to 15°, and hip flexion increased from 0° to 5° under knee extension (Table 4). The muscle strength of the right lower limb muscles around the hip joint increased slightly, with the adductor muscle increasing from MMT grade 1 to 1+ and the flexor muscle from MMT grade 1 to 1+ (Table 5). The pain was relieved, the NRS score of right inguinal region pain decreased from 5/10 to 3/10 and hip flexion and knee flexion decreased from 7/10 to 4/10 (Table 6). The overall score of DEMMI increased from 0 to 74 (Table 7). The MBI score increased from 35 to 80, and the ADL improved to basic self-care life (Table 8), indicating that she was likely to be discharged to community living.

Overall, after 2 weeks of rehabilitation, the patient was satisfied with the 2-week intervention plan and the clinical effects. She achieved significant relief of edema and pain, which were embodied in the reduced lower limb muscle circumference and NRS. The mobility and ability of daily activities was also significantly improved, as shown by the increase in DEMMI and MBI, respectively. Hip motion, hip adductor, and extensor muscle strength increased slightly. She suggested that the therapeutic exercises for hip motion and muscle strength should be begun early before the surgery.

## Discussion

**Fig. 4:**
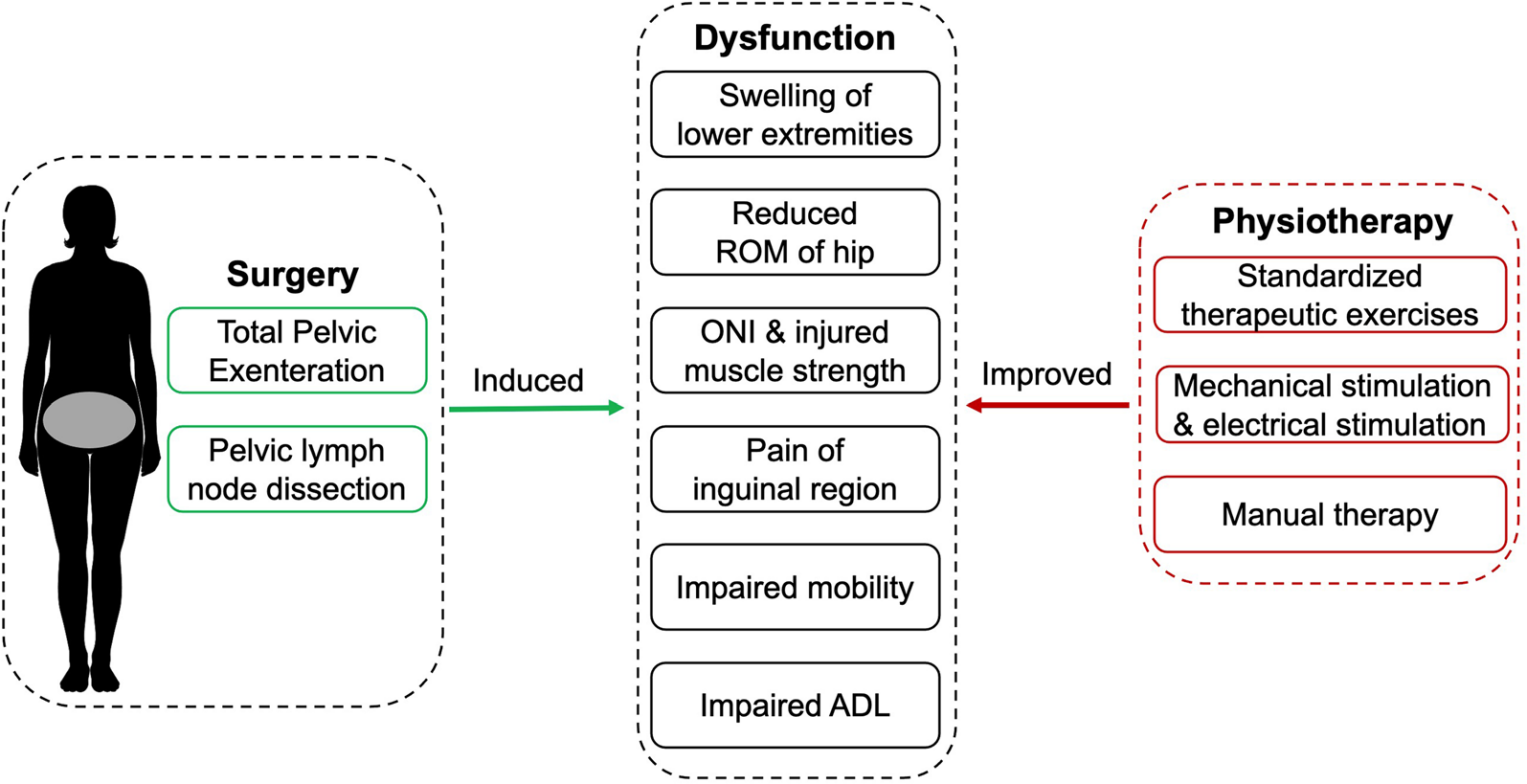
The effect of physiotherapy on dysfunction induced by pelvic surgery

This is the first case report to explore the effect of physiotherapy and mechanical stimulation on postoperative complications after TPE in a patient with cervical carcinoma. The tailor-made physiotherapy demonstrates a beneficial result for improving dysfunction induced by TPE and pelvic lymph node dissection in early postsurgical management (Fig. 4).

However, our color Doppler ultrasonography (CDS) detected left calf muscular vein thrombosis (CVT) after 1 week of physical therapy. Although anticoagulant drugs combined with physical therapy had been taken from DPO1 to reduce the risk of thrombosis, the formation of lower extremity deep vein thrombosis was still not completely avoided, which may be related to the high D-dimer and the long operative time. An expert consensus on the treatment of venous and lymphoid diseases suggested that the combination of thromboprophylactic stockings (TPS) and IPC could effectively prevent the formation of DVT after surgery (evidence level 2C); TPS pressure should be between 15 and 18 mmHg[12]. Although the combined treatment of GCS and IPC was applied in the early postoperative treatment, the pressure range of GCS was not precisely controlled, which may reduce the therapeutic effect, suggesting the need of more investigation.

After the thrombus was detected, the physiotherapist immediately stopped IPC treatment and slightly adjusted exercise therapy and manual therapy to prevent complication occurrence. For acute DVT, however, early walking and stress therapy were recommended by a previous study [13]. After 2 weeks of physical therapy, the circumference of both lower extremities decreased. Studies have shown that 30–40 mmHg pressure socks can effectively reduce lymphedema, and the maintenance effect is also long-term[14]. The muscle circumference of both lower limbs were not evaluated preoperatively and could not be compared for the patient who was referred to a physiotherapist after surgery, although the swelling was significantly reduced after treatment. Moreover, the long-term effects of swelling reduction remain unknown because of her discharge after the 2-week treatment.

The common symptoms of lower limb venous thrombosis are swelling, pain, and heaviness of the limb[15]. The American Society of Hematology (ASH) 2020 guidelines for the management of VTE suggests that the application of pressure stockings reduces pain and swelling but does not significantly reduce the risk of postthrombotic syndrome (PTS)[16]. Fortunately, the pain in the right lower limb was relieved after systematic physical therapy, which is consistent with the results of existing studies.

Obturator nerve injury (ONI) is a rare but complex complication after pelvic lymphadenectomy [17]. The most important clinical significance of ONI are adductor muscle weakness and spasm, and paresthesia in the distal medial thigh, which is consistent with this case. Bradshaw *et al*. reported that conservative methods, such as rest, physical therapy (such as ultrasound and interferential treatment), soft tissue massage, adductor muscle and pelvic strengthening exercises, oral anti-inflammatory therapy, and groin stretches, have only limited success and definitive surgical intervention is preferred[18, 19]. Gozen *et al*. reported that the preferred and most effective therapy option for ONI is an immediate repair of ONI by using microsurgical anastomosis techniques. Physiotherapy and medical treatment options are the other additive treatments of choice to improve the function of motor and/or sensation[20]. But Esther Udina *et al*. reported that, during the regeneration–reinnervation period, enhanced sensory inputs and/or motor activity by means of electrostimulation or exercise have been shown to positively influence the neuromuscular functional outcome after experimental nerve injuries in a rat, but the efficacy of the trial was only observed over 30 days[21]. Therefore, clinical experiments are needed to confirm the effect of improvement. After 2 weeks of physical therapy, the patient’s ROM of right limb had slightly improved, as indicated by an increase in active adduction from 0° to 15° and flexion from 0° to 5°. Owing to the absence of long-term follow-up, we cannot determine whether a longer duration of physical therapy leads to better improvement of mobility, or whether surgery should be considered as the first choice for such patients in the future.

Early rehabilitation activities can help patients recover ADL in the early stage after TPE surgery. After systematic physical therapy, the MBI of the patient discussed in this case increased from 35 to 80 points, and she was able to take care of herself in life. The overall score on the De Morton index increased from 0 to 74, which is consistent with existing study proposed by Jensen*et al*. [22]. The existing dysfunction for her manifested in bathing, using the toilet, and going up and down stairs, which may be related to lower limb muscle strength, especially low adductor muscle strength. The residual dysfunctions inspire us with another research direction worth thinking about, that is how to better carry out ADL training for such patients and further improve their daily activity ability.

There are three limitations in this case report: the patient rejected the preoperative assessment and exercise guidance by the physical therapists, only watched the preoperative rehabilitation education video in the ward, and performed exercises according to the video under the supervision of nurses. The therapist only treated the patient after surgery, which probably reduced the overall effect of physical therapy. It has been shown that preoperative activity level and physical health status were independent predictors of postoperative recovery and preoperative exercise therapy can be effective for reducing postoperative complication rates and length of hospital stay after abdominal surgery[23, 24]. When the patient was found to have sensory dysfunction of the medial femoral skin of the right lower limb, we did not conduct systematic sensory function training on the patient, so we could not effectively discuss the effect of physical therapy on sensory dysfunction, especially sensory dysfunction caused by obturator nerve injury.

## Conclusion

On the basis of the results, standardized physical therapy and mechanical stimulation shows an effect on the prevention and recovery of complications after TPE and pelvic lymphadenectomy. In this case, routine physical therapy significantly reduced lower limb pain and swelling and improved hip range of motion, motor function, and ADL, but still did not prevent thrombosis. This supports the necessity of multidisciplinary cooperation and early physiotherapy intervention for patients with TPE and pelvic lymphadenectomy. Further research is needed to determine the causes of thrombosis after standardized intervention and more randomized controlled trials are needed to investigate the efficacy of physical therapy after TPE.

## Data Availability

Data will not be shared because the patient in this case report does not endorse it.

## Abbreviations

ADL: Activities of Daily Life
CDS: Color Doppler ultrasonography
CVT: Calf muscular venous thrombosis
DEMMI: The de Morton Mobility Index
DVT: Deep vein thrombosis
DPO1: Day 1 postoperative
DPO7: Day 7 postoperative
DPO14: Day 14 postoperative
GCS: Graduated compression stockings
IPC: Intermittent pneumatic compression
NMES: Neuromuscular electrical stimulation
NRS: Numeric pain rating scale
MBI: The Modified Barthel Index
MLD: Manual lymphatic drainage
MMT: Manual muscle test
ONI: Obturator nerve injury
PTS: Postthrombotic syndrome
ROM: Range of motion
TPS: Thromboprophylactic stockings
TPE: Total pelvic exenteration
